# The measurement and reporting of healing-related outcomes in randomised controlled trials of compression for venous leg ulcers: a systematic review

**DOI:** 10.1186/s13063-026-09882-2

**Published:** 2026-07-17

**Authors:** Klaudia Kornalska, Chunhu Shi, Nicky Cullum, Jo Dumville

**Affiliations:** Division of Nursing, Midwifery and Social Work, School of Health Sciences, University of Manchester, Manchester, UK

**Keywords:** Compression therapy, Venous leg ulcers, Randomised controlled trials, Outcome measurement, Outcome definition, Outcome reporting, Systematic review, Wound healing, Outcome assessment

## Abstract

**Aim:**

Venous leg ulcers (VLUs) are the most prevalent type of leg ulcer and can be slow to heal. Compression therapy is a widespread, evidence-based treatment for people with VLUs, with many associated randomised controlled trials (RCTs). Healing-related outcomes are important in the field but there is currently limited insight on how these outcomes are captured and reported in RCTs. This review analyses the measurement and reporting of healing-related outcomes in RCTs of compression therapy in people with VLUs.

**Methods:**

This systematic review followed the Cochrane guidelines and is reported in line with the PRISMA statement. RCTs of compression for people with VLUs were included. Five medical literature databases were searched (September 2024), and data were extracted on the type of healing outcomes assessed, the follow-up time, the used definitions of healing-related outcomes, the wound measurement techniques, and the use and techniques of assessor masking.

**Results:**

This review included 68 RCTs, reporting 158 healing-related outcomes. These outcomes commonly evaluated change in wound size (34%) or complete wound closure (59%). Only 44% of complete wound closure outcomes were accompanied by a clear definition, and those definitions varied. Change in wound size was measured by four techniques, with planimetry used most often (51%). Notably, 56% of healing-related outcomes were evaluated by healthcare professionals and none by patients. The use of assessor masking was limited: in 38% of cases outcomes were judged by unmasked assessors, and in 42% the masking status was unclear. Follow-up duration was most commonly between 90 days and 6 months.

**Conclusion:**

This is the first systematic review to investigate the measurement and reporting of healing-related outcomes in RCTs of compression therapy for people with VLUs. Although healing-related outcomes including complete wound closure were frequently used in the included RCTs, limitations in measuring and reporting were identified. These limitations included frequent collection of surrogate outcomes, limited follow-up periods, inconsistent use of outcome definitions, variation in wound measurement techniques, and lack of assessor masking. Development of a set of guidelines on the measurement and reporting of healing-related outcomes could help to address these limitations.

**Registration:**

The protocol for this systematic review has been registered on the Open Science Framework platform and can be viewed HERE.

**Supplementary Information:**

The online version contains supplementary material available at https://doi.org/10.1186/s13063-026-09882-2.

## Introduction

Venous leg ulcers (VLUs) are the most prevalent chronic leg ulcers and arise from venous insufficiency, representing the most severe stage of venous disease [1, 2]. Although the exact cause of VLUs remains unclear, research suggests that valve dysfunction plays a crucial role in their development [1]. In healthy individuals, the valves support venous return, which means that the blood travels from the leg veins back to the heart. Valve dysfunction causes venous reflux, where the blood flows backwards, causing venous hypertension, skin fragility and leading to venous ulceration [1].

Clinically, VLUs manifest as moist, shallow wounds with irregular shapes, typically located in the gaiter leg area [3]. Owing to their chronic nature, these ulcers pose significant burdens to patients, causing pain, reduced mobility, and diminished health-related quality of life [4].

Compression therapy addresses the underlying pathophysiology of venous ulceration by enhancing venous return [5]. Compression therapy remains the cornerstone of VLU management as a treatment based on a large body of randomised controlled trial (RCTs) evidence, that includes healing-related outcomes [5].

Healing-related outcomes can be categorised into two groups: surrogate outcomes that reflect healing progress (such as a change in wound size) and the more definitive, clinical endpoint of being *healed* or complete wound closure (such as the proportion of ulcers healed at a specific time point). *Healing progress* and being *healed* are the end goal outcomes of the administered treatments. Being completely *healed* is the outcome that is most important to patients, as it often marks the end of prolonged pain, improved mobility and the ability to return to normal day-to-day activities [6].

Despite the central importance of healing-related outcomes, they are inconsistently measured and reported in RCTs of treatment for VLUs. Previous research has highlighted significant inconsistencies and a lack of transparency in how these outcomes are measured. An older systematic review of RCTs involving people with VLUs reported that healing-related outcomes were assessed at 12 different time points [7]. Another review of RCTs of chronic wound treatments reported that the terms ‘healing’ and ‘complete wound closure’ were not consistently defined across studies [8]. Moreover, a systematic review [9] reported that a 12-week median follow-up time, which is relatively short to capture complete wound healing, was frequently used in RCTs of chronic wounds [9]. The practice of masking outcome assessors to treatment allocation, a key technique for avoiding bias in outcome assessment, was also largely underreported [10].

A recent eDelphi panel study has generated a recommended core outcome set for RCTs investigating VLUs treatments [11]. Healing was one of the five recommended outcome domains (others included pain, quality of life, resource use, and adverse events), and the recommended healing outcomes were number of patients/ulcers/limbs healed, time to complete healing, and change in size of the ulcer. Although a useful indication of what should be measured in RCTs of VLU treatments, this study did not address how these outcomes should be measured and reported to ensure standardisation [11].

There is currently no consensus on how healing and complete wound closure should be defined, measured, and reported in RCTs of VLU treatments [7, 8, 12]. This lack of standardisation stands in direct contrast to well-established general guidance on RCT design and transparent reporting. Item 12 of the SPIRIT (Standard Protocol Items: Recommendations for Interventional Trials) guidelines recommend specifying the primary and secondary outcomes, including specific measurable variables, analysis metrics, methods of aggregation, and time points at the protocol development stage [13]. The CONSORT (Consolidated Standards of Reporting Trials) statement recommends that these details are also clearly explained in a final RCT report [14]. Saldanha et al. (2014), in their review of outcome specification in ophthalmology-related systematic reviews, developed a framework for completely specified outcomes. This framework included five elements, similar to the SPIRIT and CONSORT guidelines: the domain, measurement, metric, method of aggregation, and time points [15].

Despite this guidance, previous reviews have suggested that RCTs of VLU treatments often fell short of these standards, particularly in their handling of healing-related outcomes, potentially hindering the value of the RCTs’ findings for decision making [7, 8]. These previous studies, however, are over 10 years old; the current scale and scope of the variation in how healing-related outcomes are defined, measured, and reported in RCTs of VLU treatments is unclear.

This review aimed to analyse the use and reporting of healing-related outcomes in RCTs of compression therapy in people with VLUs.

## Methods

This systematic review was conducted in accordance with the Preferred Reporting Items for Systematic Reviews and Meta-Analysis (PRISMA) statement [16]. The protocol was registered with the Open Science Framework (https://osf.io/p28n6/?view_only=2b0f8a69c9044275bb5bc842861d4cdb).

### Databases and search limits

The databases were searched from inception to September 2024. The following databases were searched: the Cochrane Wounds Specialised Register; CENTRAL in the Cochrane Library; Ovid MEDLINE including In-Process and Other Non-Indexed Citations (1946); Ovid Embase (1974); and EBSCO CINAHL Plus (1937). No time limit on the date of study publication was included.

The search strategies were developed based on those used for the related Cochrane reviews, for example Shi et al. [17]. A search strategy for CENTRAL was developed and it was adapted to search the remaining databases. The Ovid MEDLINE and Embase searches were combined with the sensitivity- and precision-maximising version (2008 revision) of the Cochrane Highly Sensitive Search Strategy for identifying RCTs [18]. The CINAHL Plus search was combined with the filter developed by the Scottish Intercollegiate Guidelines Network [19]. The searches were limited to English language-only studies. All the search strategies are available in Additional file 1.

### Study inclusion/exclusion criteria

#### Types of studies included

Any published or unpublished RCTs of compression therapy (compared with standard of care, placebo, or alternative types of compression) were included. Compression was defined as a leg-compressing treatment designed to promote healing by improving venous return [20]. All types of compression, including intermittent pneumatic compression devices, were included.

RCTs were considered ‘randomised’ if randomisation was referred to in the methods section. If the description of randomisation was specified, the mention of randomisation in the report’s title or abstract was accepted as long as there was no additional information suggesting that the study was not a true RCT. Pilot studies that employed randomisation methods as described above but with a smaller population and/or shorter duration were also eligible for inclusion.

The following types of studies were excluded: feasibility studies without randomisation; trials that employed quasirandom allocation, such as alternation, allocation by date of birth or case record number; cluster RCTs where wounds on individual participants were clustered; conference abstracts, as they provided limited information for data extraction; reports of cost-effectiveness studies using RCT data; phase I RCTs where the effectiveness of intervention was not an outcome measure or the RCT included healthy participants; animal studies; diagnostic studies; studies published as theses; and letters to editors.

#### Types of participants

Studies recruiting people of any age from any care setting with active venous leg ulceration were included. As the method of diagnosis of venous ulceration can vary between studies, no standard definition was applied, and the authors’ definitions were accepted unless any criteria used seemed clinically different from what would be expected.

#### Outcomes

This review focused on healing-related outcomes, defined as any outcome describing healing progress, such as complete wound closure or changes in wound size. This review included any healing-related outcomes that were measured via any approach, analysed in any metric, and followed up for any length of time. Other outcomes, such as pain or infection, were not considered.

### Study selection process

All titles and abstracts of the identified studies were independently screened by two reviewers (KK and CS). Studies that did not meet the eligibility criteria were excluded. The full-text articles for the included abstracts were obtained and again screened for eligibility following the same process of independent screening. Any disagreements were resolved through discussion or by obtaining input from a third reviewer (JD or NC).

### Data extraction and coding

Information was extracted into a coding sheet developed in Microsoft Excel® [21], which was piloted, and standardised using 10 studies before the full data extraction. One reviewer (KK) categorised the extracted data which were checked by the second reviewer (CS). Disagreements were resolved by discussion or by obtaining input from a third reviewer (JD or NC).

#### Healing-related outcomes

**Fig. 1:**
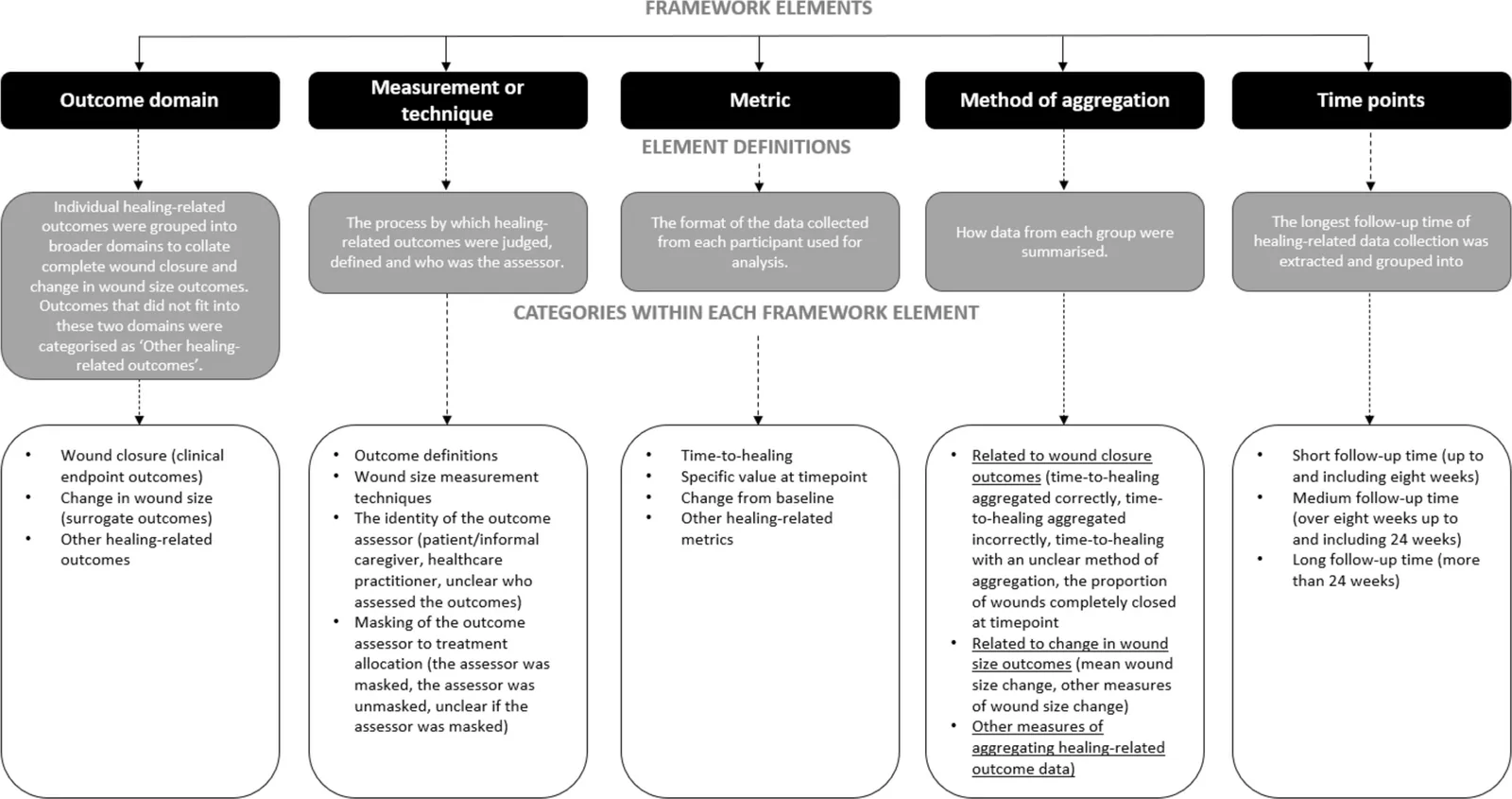
The healing-related outcomes framework used to describe how outcomes were defined, measured and reported

Extraction and coding used an adapted version of an existing outcome prespecification framework [15] (Fig. 1). Adaptation was necessary due to the complex nature of healing-related outcomes. The healing-related outcomes were organised into the following elements of the framework: outcome domain (included clinical endpoint of complete wound closure and surrogate outcome of change in wound size), measurement or technique, metric, methods of aggregation, and time points. The relevant information for each framework element was coded into specific categories, as outlined in Fig. 1.

Technical definitions were applied to the categories within the method of aggregation due to their statistical complexity (Additional file 2).

The methods of aggregation of the time-to-healing were segregated into three categories:

- Time-to-healing aggregated correctly
  The time the VLUs in the studies took to achieve complete wound closure was aggregated via a sound statistical approach. On the basis of the literature, two approaches were considered ‘sound’ [22]:
  1. The median number of days the wounds in the study took to achieve complete wound closure was reported for a sample where at least 50% of the wounds achieved complete wound closure.
  2. The mean number of days the wounds in the study took to achieve complete wound closure when 100% of wounds of those randomised achieved complete wound closure.
- Time-to-healing aggregated incorrectly
  The time the ulcers in the study took to achieve complete wound closure was incorrectly aggregated. Incorrect aggregation included the mean time, where not all wounds achieved complete wound closure, or the median time, where fewer than 50% of the wounds achieved complete wound closure [22].
- Time-to-healing with unclear aggregation
  The RCT authors did not explicitly state how time-to-healing data were aggregated, and it was not possible to infer from the results section.

Definitions were extracted verbatim and grouped on the basis of the considered factors, e.g. definitions considering the absence of scab, definitions considering epithelialisation and definitions considering complete skin closure.

#### Study and participant information

The following information was extracted via prespecified definitions (Additional file 3): the design of the RCT; the number of healing-related outcomes reported; the use of healing-related outcomes as primary or secondary outcomes; the number of study participants; and the number of participants with missing healing-related data.

### Data analysis

Information about the study design, number of participants, number of healing-related outcomes, and details about how these outcomes were measured and reported, as presented in Fig. 1, was summarised descriptively (count, proportion, median, and interquartile range (IQR)). The median number of study participants was calculated based on the number of participants recruited into each included RCT (excluding studies where only study protocol was available). The median number of healing-related outcomes was calculated based on the number of healing-related outcomes measured in each included RCT. The statistical analyses were completed using Microsoft Excel® [21].

## Results

**Fig. 2:**
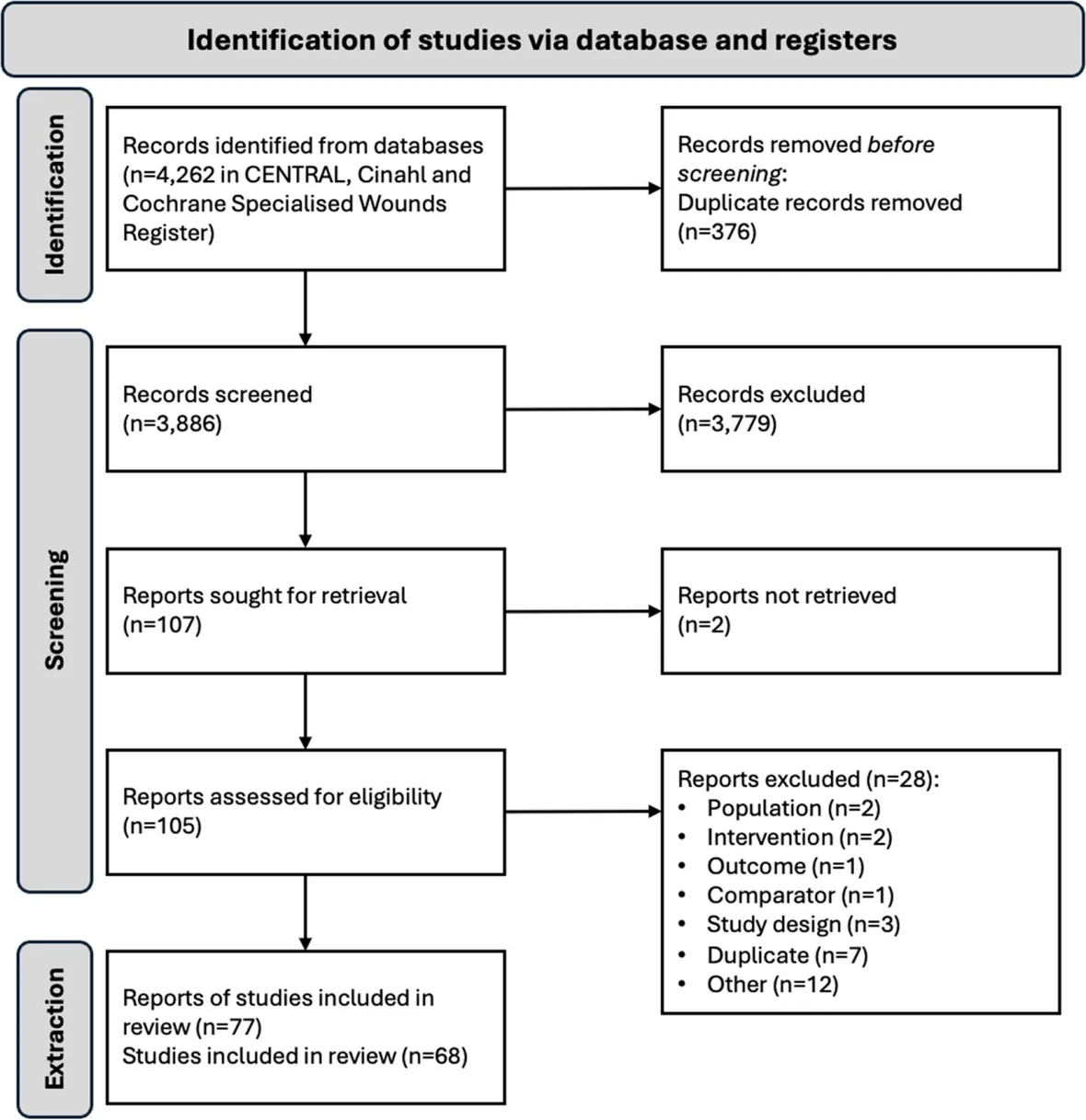
PRISMA flowchart of study selection

After screening 4262 titles and abstracts, 105 full-text reports were assessed for eligibility. In total, 77 reports describing 68 individual RCTs were included, from which data were extracted and synthesised (Fig. 2) [23–99].

The characteristics of the included RCTs are presented in Table 1. The list of all included studies is presented in the Additional file 4. The most common type of RCT design was parallel. The median number of participants across the included RCTs was 81 (IQR 47.5–128.5).

**Table 1:** Characteristics of the included RCTs (*n* = 68)

Study characteristic	% (*n*)
Total number of participants (range)^a^	6766 (12–457)
Median number of participants (IQR)	81 (47.5–128.5)
Total number of healing-related outcomes (range)	158 (1–7)
Median number of healing-related outcomes (IQR)	2 (1–3)
Study design	
Parallel design	91% (*n* = 62)
Crossover design	3% (*n* = 2)
Factorial design	1% (*n* = 1)
Mixed design	1% (*n* = 1)
The design was unclear	3% (*n* = 2)

^a^Six reports were protocols, and the number of study participants was not reported

*IQR* interquartile range

Across the 68 included RCTs, 158 instances of healing-related outcomes were reported (Table 1). When classified by outcome domain, 59% (*n* = 94) of the outcomes were complete wound closure (clinical endpoint), 34% (*n* = 53) were changes in wound size (surrogate outcomes), and 7% (*n* = 11) were other healing-related outcomes.

Among the 68 RCTs, 51% (*n* = 35 RCTs) used complete wound closure as their primary outcome, 31% (*n* = 21 RCTs) used change in wound size, and 1% (*n* = 1 RCT) used other healing-related outcomes (Table 2).

**Table 2:** Hierarchy of healing-related outcomes in RCTs of compression therapy (*n* = 68 RCTs)^a^

Type of outcomes	Primary % (*n*)	Secondary % (*n*)	Unclear % (*n*)
Complete wound closure only	28% (*n* = 19)	15% (*n* = 10)	15% (*n* = 10)
Change in wound size only	7% (*n* = 5)	0% (*n* = 0)	1% (*n* = 1)
Complete wound closure AND change in wound size	21% (*n* = 14)	16% (*n* = 11)	16% (*n* = 11)
Other^b^ AND change in wound size	3% (*n* = 2)	0% (*n* = 0)	1% (*n* = 1)
Other^b^ AND complete wound closure	3% (*n* = 2)	3% (*n* = 2)	1% (*n* = 1)
All three domains	0% (*n* = 0)	1% (*n* = 1)	4% (*n* = 3)
Other healing-related outcomes^b^ only	1% (*n* = 1)	0% (*n* = 0)	0% (*n* = 0)

^a^Some studies were double-counted, as they may have included a combination of different outcomes at different positions

^b^Other healing-related outcomes included: degree of healing measured with the RESVECH 2.0 scale; wound healing (defined as linear healing rate); ulceration area: healing dynamics; change in the Clinic, Etiologic, Anatomical, Pathophysiologic (*CEAP*) score; degree of scarring measured with the RESVECH 2.0 scale; association of clinical factors with healing and non-healing; failure to heal; effect of therapies on time and healing measured on a Likert scale; healing process (Gilman index); ulcer bed appearance; and skin condition

### How ‘complete wound closure’ and ‘change in wound size’ outcomes are measured

Of the 94 complete wound closure outcomes reported, only 44% (*n* = 41) of the outcomes in 24 RCTs were described as having been judged against a clear outcome definition. Six different definitions of complete wound closure were identified (Table 3). Full epithelialisation was part of all these definitions.

**Table 3:** Definitions of ‘complete wound closure’

Element of definition	Frequency of individual outcome (*n* of studies; %)
Full/complete/100% epithelialisation only	63% (*n* = 15/24)
Epithelialisation AND absence of scab	13% (*n* = 3/24)
Epithelialisation AND absence of exudate	4% (*n* = 1/24)
Epithelialisation OR absence of drainage	4% (*n* = 1/24)
Epithelialisation AND absence of scab AND no dressing needed	8% (*n* = 2/24)
Epithelialisation AND absence of scab AND absence of drainage	8% (*n* = 2/24)

Studies reported the techniques used to measure wound size for 87% (*n* = 46/53) of the changes in wound size outcomes. Four methods for measuring wound size were identified (Table 4), with some studies using a combination of methods. Planimetric methods were used most frequently (51%; *n* = 27/53).

**Table 4:** Methods for measuring change in wound size

Measurement methods	% of outcomes (*n*)
Planimetry methods	51% (*n* = 27/53)
Digital planimetry	56% (*n* = 15/27)
Digital planimetry (specified as the Visitrak™ system)	37% (*n* = 10/27)
Computerised scanning method	4% (*n* = 1/27)
Mechanical planimetry	4% (*n* = 1/27)
Tracing methods	19% (*n* = 10/53)
Tracing–method not described	80% (*n* = 8/10)
Tracing-method not described	60% (6/10)
Tracing; size measured by ruler	20% (2/10)
Tracing on acetate; manual square count	10% (*n* = 1/10)
Tracing on acetate; weight of acetate paper	10% (*n* = 1/10)
Wound imaging techniques	4% (*n* = 2/53)
Wound imaging software	50% (*n* = 1/2)
Wound imaging using the Silhouette System™	50% (*n* = 1/2)
A combination of methods	11% (*n* = 6/53)
Computerised planimetry of tracings of the ulcer perimeter made onto transparent acetate	17% (*n* = 1/6)
Tracing on acetate combined with wound depth measure (digital calliper with a depth gauge)	17% (*n* = 1/6)
Planimetry combined with photographs	17% (*n* = 1/6)
Tracing combined with planimetry	32% (*n* = 2/6)
Square grids combined with wound imaging	17% (*n* = 1/6)
Wound grid device	2% (*n* = 1/53)
Methods unclear	13% (*n* = 7/53)

### Metrics and methods of aggregation

Four different types of metrics were identified (Table 5). The specific value at a timepoint was the most frequently used metric for the complete wound closure domain (57% of outcomes; *n* = 54/94). The change from baseline was the only metric used in the change in wound size domain (100% of outcomes; *n* = 53/53).

**Table 5:** Metrics and methods of aggregation of healing-related outcomes in RCTs of compression therapy

Domain	Metric % (*n*)	Method of aggregation % (*n*)
Complete wound closure (*n* = 94)	Time-to-healing (42%; *n* = 39/94)	Time-to-healing (correctly aggregated) (70%, *n* = 27/39)
		Time-to-healing (incorrect) (15%; *n* = 6/39)
		Time-to-healing (unclear) (15%; *n* = 6/39)
	Specific value at timepoint (57%; *n* = 54/94)	Proportion of wounds completely closed at timepoint (96%; *n* = 52/54)
		Unclear method of aggregating complete wound closure (2%; *n* = 1/54)
		Other methods of aggregating complete wound closure (2%; *n* = 1/54)
	Change from baseline (1%; *n* = 1/94)	Proportion of wounds completely closed at timepoint (100%; *n* = 1/1)
Change in wound size (*n* = 53)	Change from baseline (100%; *n* = 53/53)	Mean and median wound size change (4%; *n* = 2/53)
		Mean wound size change (53%; *n* = 28/53)
		Median wound size change (13%; *n* = 7/53)
		Other methods of aggregating change in wound size (6%; *n* = 3/53)
		Unclear method of aggregating change in wound size (24%; *n* = 13/53)
Other healing-related outcomes (*n* = 11)	Change from baseline (55%; *n* = 6/11)	Median wound size change (17%; *n* = 1/6)
		Other method of aggregation (17%; *n* = 1/6)
		Unclear method of aggregation (66%; *n* = 4/6)
	Specific value at timepoint (9%; *n* = 1/11)	Unclear method of aggregation (100%; *n* = 1/1)
	Other metrics (36%; 4/11)	Other method of aggregation (25%; *n* = 1/4)
		Unclear method of aggregation (75%; *n* = 3/4)

Four different types of data aggregation were identified in the complete wound closure domain, five in the change in wound size domain, and three in the other domain (Table 5). For the complete wound closure domain, the proportion of wounds completely closed at a timepoint was most frequently used (55% of outcomes; *n* = 52/94). Notably, 6% of complete wound closure outcomes (*n* = 6 outcomes) were incorrectly aggregated for time-to-healing. For the change in wound size domain, the mean change in wound size (53% of outcomes; *n* = 28/53) was most frequently used, with 24% (*n* = 13) of outcomes being unclear on the aggregation technique used.

### Outcome assessors

In total, 56% (*n* = 88/158) of the healing-related outcomes reported in the RCTs were assessed by a healthcare professional, and no healing-related outcome was reported as being assessed by the patient or caregiver. The outcome assessor was not reported for 44% (*n* = 70) of the outcomes.

**Fig. 3:**
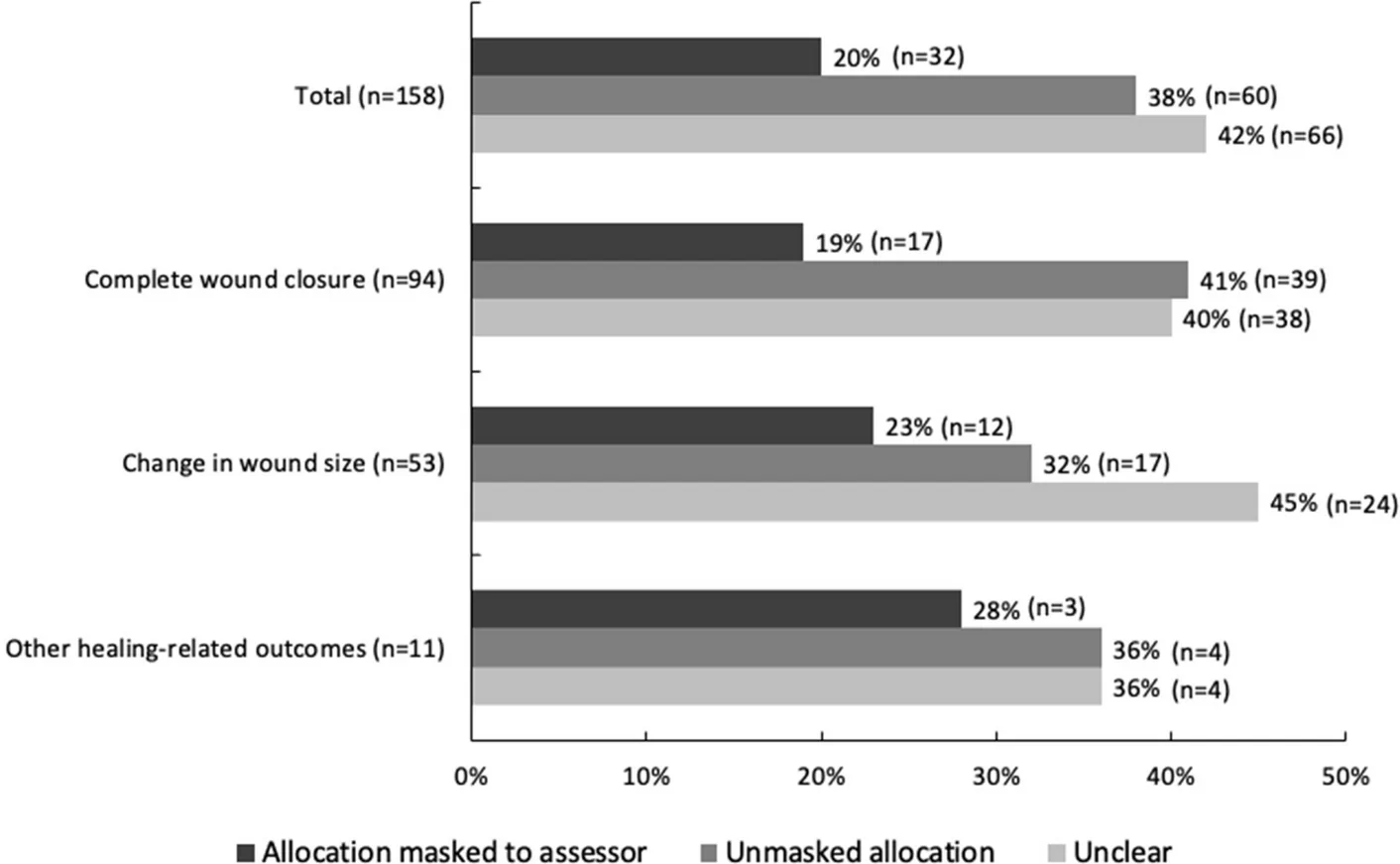
Outcome assessment in the presence of masking to treatment allocation (158 outcomes, 16 studies)

Twenty percent (*n* = 32/158) of the outcomes across 16 RCTs were reported by an assessor masked to treatment allocation, and 38% (*n* = 60/158) of the outcomes were evaluated unmasked. It was unclear from the study report whether outcome assessors were masked to treatment allocation for 42% (*n* = 66/158) of the outcomes (Fig. 3).

For 66% of the outcomes (*n* = 2/32), how the outcome assessors were masked was not explained. Where information was available, photography (28% of outcomes; *n* = 9/32) was most commonly described. For 6% of the outcomes (*n* = 2/32), the study authors described a process of using different ‘rooms’ to separate treatment removal from the assessment process.

### Time points

A duration of follow-up between 90 days and 6 months (regarded as the ‘medium’ duration) was frequently used to assess healing-related outcomes, and this duration was similar across the different types of healing-related outcomes (Table 6). Over half of the outcome data were collected at a follow-up of up to 6 months (78%; *n* = 73/94).

**Table 6:** The longest follow-up time in the healing-related outcome domain

Follow-up time	Complete wound closure (*n* = 94) % (*n*)	Change in wound size (*n* = 53) % (*n*)	Other healing-related outcomes (*n* = 11) % (*n*)
Up to 8 weeks	2% (*n* = 2)	13% (*n* = 7)	9% (*n* = 1)
9 to 24 weeks	76% (*n* = 71)	68% (*n* = 36)	64% (*n* = 7)
More than 24 weeks	22% (*n* = 21)	19% (*n* = 10)	27% (*n* = 3)

## Discussion

For the first time, this review summarised in a systematic way how healing-related outcomes were defined, collected, and measured in RCTs of compression therapy for VLUs. Certain shortcomings were highlighted that could limit effective study interpretation and comparison. Reaching this new perspective was enabled by using and adapting the pre-existing framework by Saldanha et al. [15]. There were key emerging issues: the type of outcomes assessed in RCTs, the use of outcome definitions, the techniques used to measure ulcer size, the duration of follow-up, and the masking of outcome assessors.

### What types of healing-related outcomes were measured in RCTs

The clinical endpoint of complete wound closure was measured frequently (59% of all outcomes), whilst the surrogate outcome of change in wound size was used as the primary outcome in 31% of the included RCTs. This finding was consistent with previous research reporting that 40% of RCTs in chronic wounds used surrogate outcomes as primary outcomes [9]. The predictive value of surrogate outcomes, such as changes in wound size, is unclear.

Two systematic reviews suggested that the measure of change in wound size aggregated as a percentage of wound area reduction can be a good predictor of wound healing at 24 weeks when the percentage of wound area reduction cut-off was 40% at 4 weeks [100, 101]. Although these systematic reviews presented an interesting point of view, there are fundamental problems with the predictive value being assessed via the C-statistic or the area under the curve for receiver operating characteristic (AROC) curve [100, 101]. These two statistical techniques modelled the discriminatory (rather than predictive) value of the surrogate outcome, meaning that they assessed how well the outcome can dichotomise people into those who will or will not heal [102]. This discriminatory ability of surrogates was assessed in isolation from the treatment effect or other factors that could delay or arrest wound healing after the end of the study. The discriminatory performance of the percentage of wound area reduction varied in the included studies from poor to excellent [100, 101], further suggesting that the percentage of wound area reduction does not perform consistently as a surrogate of complete wound closure. Notably, the studies included in these systematic reviews were also at high risk of bias, mainly due to attrition and confounding factors.

Existing problems with assessing change in wound size in RCTs remain. There are multiple ways in which the change in wound size can be summarised, including percentage of wound area reduction, gross area reduction or wound margin advancement. The results of these aggregation techniques can vary depending on the wound size, potentially creating confusion on how the RCT results should be interpreted. Bull et al. (2022) also assessed these metrics in a small cohort of 40 VLU patients and reported that they follow a linear trajectory over the initial 4-week period of healing. It was not clear, however, whether VLUs follow this trajectory after the initial 4 weeks [103]. In addition, Margolis et al. (1993) suggested that even though a change in wound size could predict whether complete wound closure will occur, it does not predict how long it will take [104]. Precisely measuring the wound size presents additional challenges, such as inconsistent identification of the wound borders, prohibiting accurate and precise assessment [105].

Given the lack of robust validation of change in wound size as a surrogate outcome, following VLUs to complete closure has clear merits. Patients view complete wound closure as the most important outcome, and it is more objective than a change in wound size [6]. Complete wound healing is associated with a change in the need for treatment and reduced costs. Although complete wound closure healing-related outcomes are frequently used in RCTs, it is unclear how they are measured and defined. For example, both the identity of the assessors and how they were masked to treatment were not reported for 42% of the outcomes. This lack of transparency leads to uncertainty around the risk of bias and the validity of the conclusions of RCTs.

### How healing-related outcomes were defined, measured, and reported in RCTs

This systematic review showed that VLU RCTs frequently failed to adhere to the widely accepted standards of outcome collection and measurement [13].

First, 56% of the outcomes were not judged against a definition, and 44% did not map to a consistent definition. This is in line with the systematic review by Gethin et al. (2015), who reported that 61% of VLU RCTs lacked a definition of healing. Research has shown that the interpretation of complete wound closure can vary across different outcome assessors [106]. Defining outcomes is crucial to ensure precise, comparable and specific study results [13].

Second, there was variation in the way the change in wound size as a marker of wound healing progress was measured across studies. The most frequent techniques were digital planimetric methods (for 51% of the outcomes) and manual tracings on acetate (for 23% of the outcomes). The extent to which these different techniques yield consistent measurements is not clear. Multiple studies have shown discrepancies between planimetric and manual tracing techniques, with the disparity increasing with ulcer size [107, 108]. Rogers et al. (2010) reported that manual measurement of elliptical wounds via a ruler overestimated the wound size by 40% compared with the noncontact digital planimetric method (SilhouetteMobile) [109].

Third, the duration of follow-up varied across RCTs, with 6 months or shorter being the most frequent (78% of complete wound closure outcomes). This short duration of follow-up is concerning since research has shown that at least 6 months of follow-up is needed to detect the effects of treatment on the healing process in VLUs [12]. A series of pragmatic RCTs, the Venous Leg Ulcer Study (VenUS) I, III, III, and IV, revealed that VLUs can take a long time to heal. These RCTs reported a median time-to-healing that ranged from 92 days (CI, 71–113) in VenUS I to 245 days (CI, 166-not estimable) in VenUS II [65, 110]. This means that the follow-up times frequently used in RCTs of VLUs would not be sufficient to measure complete wound closure or persistence of healing.

Finally, masking of outcome assessors to treatment allocation was frequently not reported (66 of 158 outcomes) in RCTs and assessors were reported as unmasked (60 of 158 outcomes) [111–113]. There is some uncertainty regarding the impact of masking. Two studies by Hrobjartsson et al. (2012, 2014) reported bias associated with the lack of masking in subjective binary outcomes and time-to-event analyses [112, 113]. In contrast, Moustgaard et al. (2020), in their review of 142 meta-analyses of RCTs, reported no evidence of differences in the estimated treatment effect between studies with and without masking [114]. Masking remains recommended as a precautionary approach in RCTs. This is particularly important in areas such as wound healing, where outcomes are often subjective, and the outcome definitions and measurement techniques vary.

### Implications of findings

The way in which healing-related outcomes are defined, measured, and reported in RCTs of compression therapy for VLUs creates difficulties in the accurate interpretation and synthesis of data. It is recommended that study authors take several measures to increase the consistency and accuracy of measuring and reporting outcomes. First, engaging an experienced statistician when designing new RCTs is recommended to provide a robust approach to statistical analyses and avoid errors, such as incorrect aggregation of data. Second, following reporting guidelines, such as the CONSORT or SPIRIT statements, is crucial to ensure thorough and transparent reporting [13, 14, 115]. Specifically, CONSORT and SPIRIT offer extensions relevant to surrogate outcomes in RCTs: CONSORT-Surrogate and SPIRIT-Surrogate [116]. Although the use of surrogate outcomes in RCTs of VLUs is still debated, using these specific checklists can improve transparency, interpretation, and usefulness of findings. Third, measuring complete wound closure outcomes, instead of changes in wound size, provides additional benefits. Complete wound closure outcomes can be measured more consistently and provide a more definitive indication of healing status. To maximise the benefits of measuring complete wound closure, consistent definitions of ‘healing’ and ‘healed wounds’ need to be used. This can be operationalised through developing a set of guidelines of not only what outcomes should be measured in RCTs of VLUs but also listing the best measurement practices. Finally, although no indication was found that study participants are currently assessing their own healing, this approach could provide added value. For example, engaging patients in reporting their own wound closure outcomes could reduce the need for clinical visits, potentially reduce costs and allow for a longer follow-up time.

### Study limitations

The authors acknowledge several limitations in this review. First, the protocol was developed iteratively because of the complex methodological nature of this review. In line with best practices, the research questions and the overall analytical approach were finalised before the data analysis and interpretation began. Second, the conclusions of this review were based on published RCT reports, and it was not clear if further details would have been revealed had protocols been requested from the study authors. Finally, only English-language publications were included due to a lack of funding for translation. Although some studies of interest might have been missed, the sample of included publications represented a significant dataset.

## Conclusions

This systematic review provides new insights into the methodological limitations and lack of transparent outcome reporting in RCTs of compression therapy for VLUs. Limitations identified were frequent collection of surrogate healing-related outcomes, limited follow-up periods, inconsistent use of outcome definitions, variation in wound measurement techniques, lack of patient-reported healing-related outcomes, and infrequent assessor masking. These problems were compounded by poor reporting, preventing confident interpretation of study results and their subsequent synthesis in systematic reviews. The authors of future RCTs in this field should prioritise the careful selection, measurement, analyses and reporting of healing-related outcomes, using meaningful follow-up periods, and with adherence to conduct and reporting guidelines.

## Supplementary Information

Additional file 1. Search strategy.

Additional file 2. Glossary: technical definitions of the methods of aggregation of healing-related outcome data.

Additional file 3. Pre-specified definitions of study characteristics.

Additional file 4. List of included studies.

Additional file 5. (PRISMA_2020).

## Data Availability

The datasets used and/or analysed during the current study are available from the corresponding author on reasonable request.

## Abbreviations

AROC: Area under curve for receiver operating characteristic
CEAP: Clinic, Etiologic, Anatomical, Pathophysiologic
CONSORT: The Consolidated Standards of Reporting Trials
IQR: Interquartile range
PRISMA: The Preferred Reporting Items for Systematic Reviews and Meta-Analysis
RCT: Randomised controlled trial
SPIRIT: The Standard Protocol Items: Recommendations for Interventional Trials
VenUS: Venous leg Ulcer Study
VLU: Venous leg ulcer
