# Supplementary material for: The measurement and reporting of healing-related outcomes in randomised controlled trials of compression for venous leg ulcers: a systematic review
Source: Trials. 2026 Jul 17;27:604. doi: 10.1186/s13063-026-09882-2 (PMC13656612; doi:10.1186/s13063-026-09882-2)
Supplement: Supplementary file 1 [file 13063_2026_9882_MOESM1_ESM.docx]

Additional file 1. Search strategy

**CENTRAL search strings for venous leg ulcers and treatments (from 1996 to September 2024)**

1. MeSH descriptor: [varicose ulcer] explode all trees
2. Title, abstract, keywords: (varicose ulcer*) OR (venous ulcer*) OR (stasis ulcer*) OR (crural ulcer*) OR (ulcus cruris) OR (hypertension ulcer*) OR (venous leg ulcer*) OR (venous ulceration)
3. 1 OR 2
4. MeSH descriptor: [compression bandage] explode all trees
5. Title, abstract, keywords: (compression OR bandag* OR stocking* OR hosiery OR wrapp*)
6. 4 OR 5
7. 3 AND 6

**Ovid MEDLINE including In-Process & Other Non-Indexed Citations search strings for venous leg ulcers and treatments (from 1946 to September 2024)**

1. Exp varicose ulcer/
2. (varicose ulcer$ OR venous ulcer$ OR stasis ulcer$ OR crural ulcer$ OR ulcus cruris OR hypertension ulcer$ OR venous leg ulcer$ OR venous ulceration).ti,ab.
3. 1 OR 2
4. exp Stockings, Compression/
5. (compression OR bandag$ OR stocking$ OR hosiery OR wrapp$).ti,ab.
6. 4 OR 5
7. 3 AND 6

**Cochrane Highly Sensitive Search Strategy for RCTs**

1. randomized controlled trial.pt. OR controlled clinical trial.pt. OR randomized.ab. OR placebo.ab. OR clinical trials as topic.sh. OR randomly.ab. OR trial.ti.
2. exp animals/ NOT humans.sh.
3. 1 NOT 2

**Embase search strings for venous leg ulcers and treatments (from 1974 to September 2024)**

1. Exp varicose ulcer/
2. (varicose ulcer* OR venous ulcer* OR stasis ulcer* OR crural ulcer* OR ulcus cruris OR hypertension ulcer* OR venous leg ulcer* OR venous ulceration).ti,ab.
3. 1 OR 2
4. exp Compression Therapy/
5. (compression OR bandag* OR stocking* OR hosiery or wrap*).ti,ab.
6. 4 OR 5
7. 3 AND 6

**CINAHL Plus search strings for venous leg ulcers and treatments (from 1937 to September 2024)**

1. (MH "Venous Ulcer+") OR (“venous ulcer*” OR "venous leg ulcer*" OR “varicose ulcer*” OR “stasis ulcer*” OR “Crural ulcer*” OR “Ulcus cruris*” OR “Hypertension ulcer*” OR “Venous ulceration*”)
2. (MH “Compression Therapy+”) OR (compression OR bandag$ OR stocking$ OR hosiery OR wrapp$)
3. 1 AND 2

**SIGN filter for RCTs**

1. TX allocat* random* OR (MH "Quantitative Studies") OR (MH "Placebos") OR TX placebo* OR TX random* allocat*OR (MH "Random Assignment") OR TX randomi* control* trial* OR TX ( (singl* n1 blind*) or (singl* n1 mask*) ) OR TX ( (doubl* n1 blind*) or (doubl* n1 mask*) ) OR TX ( (tripl* n1 blind*) or (tripl* n1 mask*) ) OR TX ( (trebl* n1 blind*) or (trebl* n1 mask*) ) OR TX clinic* n1 trial* OR PT Clinical trial OR (MH "Clinical Trials+")
