# Supplementary material for: The measurement and reporting of healing-related outcomes in randomised controlled trials of compression for venous leg ulcers: a systematic review
Source: Trials. 2026 Jul 17;27:604. doi: 10.1186/s13063-026-09882-2 (PMC13656612; doi:10.1186/s13063-026-09882-2)
Supplement: Supplementary file 2 [file 13063_2026_9882_MOESM2_ESM.docx]

Additional file 2. Glossary: technical definitions of the methods of aggregation of healing-related outcome data

| Method of aggregation | Technical definition |
| --- | --- |
| Related to complete wound closure outcomes | |
| Time-to-healing aggregated correctly | Time the ulcers in the studies took to achieve complete wound closure was aggregated using a sound statistical approach. Based on the literature, I considered two approaches to aggregation as ‘sound’ (1):  The median number of days the wounds in the study took to achieve complete wound closure reported for a sample where at least 50% of the wounds achieved complete wound closure.  The mean number of days the wounds in the study took to achieve complete wound closure when 100% of wounds of those randomised achieved complete wound closure. |
| Time-to-healing aggregated incorrectly | The time the ulcers in the study took to achieve complete wound closure was aggregated incorrectly. An incorrect aggregation includes mean time, where not all wounds achieve complete wound closure, or median time where fewer than 50% of wounds achieved complete wound closure (1). |
| Time-to-healing with unclear aggregation | The RCT authors did not explicitly state how time-to-healing data were aggregated and it was not possible to infer from the results section. |
| Other methods of aggregation | The method of aggregation did not fit into any of the categories above. |
| Related to change in wound size outcomes | |
| Proportion of wounds completely closed at timepoint | The complete wound closure was of interest and observed at a specified timepoint (e.g., percentage of wound closed at the last follow-up). |
| Mean wound size change | The reduction in wound size from baseline was aggregated as a mean (e.g., mean size of wound reduction in cm^2^). |
| Median wound size change | The reduction in wound size from baseline was aggregated as a median (e.g., median percent change in wound size area). |
| Not related to either complete wound closure or change in wound size | |
| Other wound size change | Change in wound size is aggregated in a different way, other than mean or median (e.g., percentage of area reduction). |

**References:**

1.	Schober P, Vetter TR. Survival Analysis and Interpretation of Time-to-Event Data: The Tortoise and the Hare. Anesth Analg. 2018;127(3):792-8.
