# Supplementary material for: The measurement and reporting of healing-related outcomes in randomised controlled trials of compression for venous leg ulcers: a systematic review
Source: Trials. 2026 Jul 17;27:604. doi: 10.1186/s13063-026-09882-2 (PMC13656612; doi:10.1186/s13063-026-09882-2)
Supplement: Supplementary file 3 [file 13063_2026_9882_MOESM3_ESM.docx]

Additional file 3. Pre-specified definitions of study characteristics

**Study Design**

The design features of the included RCTs will be coded as parallel, crossover, factorial and/or cluster RCTs (1). When different design features are combined in one research study (e.g., parallel factorial design), this will be recorded as a mixed design RCT study with different aspects recorded (e.g., mixed RCT study design: parallel crossover design).

First, the publication will be scrutinised for design features as defined by the authors. If the authors did not specify the exact design; however, the design is narratively described in the methods section, the design will be coded on the basis of the definitions below:

- Parallel RCTs – Studies where participants are randomised between two or more RCT arms and participants in each study arm receive a different intervention. The participants are expected to stay in their assigned group for the duration of the study (1).
- Crossover RCTs – Studies that assess two or more interventions and participants are randomly assigned to different study arms; however, they later switch at a prespecified time to another treatment group (often with a washout period between treatments). Each participant receives all possible treatments under investigation; however, the order in which the treatments are received is randomly assigned (1).
- Factorial RCTs—Like crossover RCTs, factorial RCTs are studies that assess two or more interventions simultaneously; however, participants do not switch their treatment groups and are anticipated to remain in their assigned treatment group until the end of the study (2). For example, participants are randomised to either treatment A or not A and to Treatment B or not B. This results in four treatment groups: A + B, A alone, B alone, and neither A nor B (akin to two RCTs in one) (2).
- Cluster RCT – Studies where the unit of randomisation is not individual participants but those clustered in some way (e.g., entire hospitals, clinics, GPs) (3). In RCTs of venous leg ulcer cluster, RCT designs can be used where an individual is the ‘cluster’ and where individual wounds are the units of randomisation.

**Missing data**

The number of participants randomised for which missing data were reported in the RCT will be recorded as stated by the RCT authors. However, if the number of participants with missing data is not explicitly stated by the RCT authors, the number of participants with missing data will be estimated via the following formula:

*N of participants with missing data=N of participants randomised - N of participants analysed*

Where possible, the volumes of missing data will be reported on a healing-related outcome basis.

**References:**

1.	Nair B. Clinical Trial Designs. Indian Dermatol Online J. 2019;10(2):193-201.

2.	Couper D, Hosking J, Cisler R, Gastfriend D, Kivlahan D. Factorial designs in clinical trials: options for combination treatment studies. Journal of Studies on Alcohol Supplement. 2005;66.

3.	Rethink Clinical Trials. SECTION 3: Cluster Randomized Trials 2021 [
