# Supplementary material for: The measurement and reporting of healing-related outcomes in randomised controlled trials of compression for venous leg ulcers: a systematic review
Source: Trials. 2026 Jul 17;27:604. doi: 10.1186/s13063-026-09882-2 (PMC13656612; doi:10.1186/s13063-026-09882-2)
Supplement: Supplementary file 4 [file 13063_2026_9882_MOESM4_ESM.docx]

Additional file 4. List of included studies

| **Study** | **RCT design** | **N** | **Verbatim healing-related outcome** | **Outcome assessor** | **Masking** | **Follow-up time** |
| --- | --- | --- | --- | --- | --- | --- |
| Ashby et al. 2014 and Ashby et al. 2014 (short report) (1) | Parallel | 457 | - Time-to-healing (masked) in days from randomisation - Time-to-healing (unmasked) in days from randomisation | HCP | Yes (primary time-to-healing outcome); No (secondary time-to-healing outcome) | 12 months |
| Moody et al. 1999 (2) | Parallel | 52 | - Leg ulcer healing: Average percentage reduction in the wound bed surface area - Leg ulcer healing: Number of wounds healed in 12 weeks - Leg ulcer healing: Average time-to-healing (Incorrect) | HCP | Unclear from the study description | 12 weeks |
| Folguera-Alvarez et al. 2016 (3) | Parallel | NR (protocol only) | - Complete healing: Time elapsed between start of the study and complete healing of the wound (in days) - Degree of healing: RESVECH 2.0 scale - Complete healing: incidence rates of ulcers with complete healing | HCP | Yes | 12 weeks |
| NCT00534937 (4) | Parallel | 89 | - Complete healing rate - Change in wound surface area for nonhealed subjects - Percentage Change in Volume of the Affected Limb - Time-to-healing of the Venous Stasis Ulcer | Unclear from the study description | No | 12 weeks |
| Rowland et al. 2000 (5) | Crossover | 16 | - Ulcer size in cm^2^ - Complete healing | Ulcer size reported by HCP;  unclear for other outcomes | Unclear from study description | 6 months |
| NCT02680834 (6) | Parallel | 56 | - Percentage of venous leg ulcer area reduction | Unclear from study description | No | 16 weeks |
| NCT02561013 (7) | Parallel | NR (protocol only) | - Number of ulcers achieving complete closure - Wound area reduction and relative wound area reduction - Relative wound area reduction ≥ 40% at 4 weeks - Time to complete closure | Unclear from study description | Unclear from study description | 12 weeks for all outcomes except relative wound area reduction ≥40% which was measured at 4 weeks |
| Dolibog et al. 2014 (8) | Parallel | 147 | - Total ulcer surface area in cm^2^ - Length and width in cm - Volume in cm^3^ | HCP | Yes | 2 months |
| Scriven et al. 1998 (9) | Parallel | 53 | - Ulcer area in cm^2^ - Ulcer healing | Unclear from study description | Unclear from study description | Unclear from study description |
| Moffat et al. 2008 (10) | Crossover | 81 | - Wound healing: percent of wounds that healed - Wound healing: median percent change in wound area - Wound healing: linear healing rate | HCP | No | 8 weeks |
| Szewczyk et al. 2010 (11) | Parallel | 46 | - Ulceration area: healing dynamics - Ulceration area: percentage of area loss - Change in CEAP scoring - Ulceration area: number and percentage of healed ulcers | HCP | Unclear from study description | 12 weeks |
| Brizzio et al. 2010 (12) | Parallel | 55 | - Proportion of healing within 90 days - Proportion of healing within 180 days - Time-to-healing | HCP | No | 90 days: proportion to healing;  180 days: proportion to healing and time-to-healing |
| de Abreu et al. 2015 (13) | Unclear | 19 | - Reduction of the area in cm^2^ | HCP | Unclear from study description | 13 weeks |
| Gillett et al. 2019 (14) | Parallel | 92 | - Percentage of ulcers completely healed at week 16 - Percentage of healed patients at weeks 4, 8 and 12 - Complete healing time (mean) | HCP | No | 16 weeks: percentage of ulcers completely healed and complete healing time;  12 weeks: percentage of healed patients |
| Milic et al. 2010 (15) | Parallel | 131 | - Healing of the VLU at week 26 - Time to heal | HCP | Unclear from study description | 26 weeks |
| Weller et al. 2012 (16) | Parallel | 45 | - Percentage reduction of wound size from baseline to week 12 (end of treatment) - (Absolute) reduction in ulcer size - Time to complete healing - Proportion of ulcers healed | HCP | No | 12 weeks |
| Finlayson et al. 2014 (17) | Parallel | 103 | - Healed ulcer (not further described) - Mean percentage reduction of ulcer area | HCP | Unclear from study description | 24 weeks |
| Mosti et al. 2020 (18) | Parallel | 66 | - Ulcer healing rate - Ulcer size reduction | HCP | Unclear from study description | 12 weeks |
| Franks et al. 2004 (19) | Mixed design | 156 | - Ulcer closure | Unclear from study description | Unclear from study description | 24 weeks |
| O'Brien et al. 2003 (20) | Parallel | 200 | - Time to heal | HCP | Unclear from study description | 12 weeks |
| Milic et al. 2007 (21) | Parallel | 150 | - Complete ulcer healing | Unclear from study description | Unclear from study description | 500 days |
| Junger et al. 2004 (22) | Parallel | 121 | - Rate of complete ulcer healing (Gilman index) - Time to complete healing | HCP | Yes (rate of complete ulcer healing); No (time to complete healing) | 12 weeks |
| NCT00558662 (23) | Parallel | 234 | - Venous leg ulcer healing - Wound size reduction | Unclear from study description | Yes | 12 weeks |
| NCT02364921 (24) | Parallel | 93 | - Degree of scarring (RESVECH 2.0 scale) | Unclear from study description | Yes | 12 weeks |
| NCT02782689 (25) | Parallel | 92 | - Percentage of patients with complete wound closure | Unclear from study description | No | 16 weeks |
| NCT02790593 (26) and Stather et al. 2021 (27) | Parallel | 40 | - Time-to-healing - Percentage healed - Surface area measurement | Unclear from study description | No | 24 weeks |
| Polignano et al. 2004 (28) | Parallel | 68 | - The proportion of ulcers healing - Time-to-healing - Ulcer area (estimated as length x width) | Unclear from study description | Unclear from study description | 24 weeks |
| Polignano et al. 2004 (29) | Parallel | 56 | - Proportion of ulcers healed - Time-to-healing - Differences in time-to-healing between the two comparative groups | HCP | Unclear from study description | 12 weeks |
| Junger et al. 2004 (30) | Parallel | 191 | - Complete healing - Time-to-healing | HCP | No | 12 weeks |
| Mariani et al. 2008 (31) | Parallel | 60 | - Ulcer healing - Associations of various clinical factors between each other, with healing or nonhealing and time-to-healing | Unclear from study description | No | 4 months |
| Meyer et al. 2002 (32) | Parallel | 112 | - Complete ulcer healing - Time taken to heal (mean time compared between the groups) - Time taken to heal (mean) - Failure to heal within 26 weeks | Unclear from study description | No | 26 weeks |
| Ukat et al. 2003 (33) | Parallel | 89 | - Complete healing of the study ulcer within 84 days - Time-to-healing | HCP | Unclear from study description | 12 weeks |
| Moffat et al. 2003 (34) | Parallel | 112 | - Time to complete closure | HCP | No | 24 weeks |
| Moffat et al. 1999 (35) | Parallel | 233 | - Healing rates (not further specified) - Time-to-healing | Unclear from study description | No | 24 weeks |
| Harrison et al. 2011 (36) and NCT00202267 (37) | Parallel | 424 | - Time (weeks) to complete healing - Rate of reduction in ulcer area - 4 weeks or greater improvement in time-to-healing | HCP (time to complete healing); Unclear for other outcomes | Yes (time to complete healing); Unclear for other outcomes | 14 months |
| Morrell et al. 1998 (38) | Unclear from study description | 233 | - Time to complete healing of all ulcers within the 12 month follow up | HCP | Unclear from study description | 12 months |
| DePalma et al. 1999 (39) | Parallel | 38 | - Time-to-healing (mean) - Rate of area decrease - Rate of percentage area decrease - Linear (planimetric) healing rate | HCP | Unclear from study description | 12 weeks |
| Gould et al. 1998 (40) | Parallel | 39 | - Wound healing (a progression of quality in ulcer outcome from ‘no improvement or deterioration’ through ‘improved but did not heal’ to ‘healed during trial’) - Time-to-healing | HCP | Yes | 16 weeks |
| Hendrics et al. 1985 (41) | Parallel | 21 | - Healing time | HCP | Unclear from study description | 78 weeks |
| Iglesias et al. 2004 (42) and Nelson et al. 2004 (43) | Parallel | 387 | - Complete healing of all the ulcers on the trial leg - Proportion of patients healed at 12 and 24 weeks | HCP | Yes | 24 weeks |
| Nikolovska et al. 2004 (44) | Parallel | 104 | - Complete healing of the reference ulcer (n and percentage) - Time-to-healing (median) - Mean rate of healing per day (cm^2^) | HCP | Yes | 6 months |
| McCulloch et al. 1994 (45) | Parallel | 22 | - Complete healing - Healing rates per day (cm^2^) | HCP | Unclear from study description | 6 months |
| Taradaj et al. 2009 (46) | Parallel | 80 | All measured by Gilman index and relative change):   - Healing process - Complete healing - Relative change of the total surface area - Relative change of length - Relative change of width - Relative change of volume | Unclear from study description | No | 26 weeks |
| Partsch et al. 2001 (47) | Parallel | 116 | - Complete healing | Unclear from study description | Unclear from study description | 16 weeks |
| Harley et al. 2004 (48) | Parallel | 30 | - Healing status (number of patients who were healed after treatment) - Healing duration: the average number of days a patient spent under treatment | HCP | Unclear from study description | 30 months |
| Travers et al. 1992 (49) | Parallel | 27 | - Rate of venous ulcer healing | HCP | Unclear from study description | 7 weeks |
| Wilkinson et al. 1997 (50) | Parallel | 29 | - Healing (binary outcome: healed or not healed) | HCP | No | 12 weeks |
| Taylor et al. 1998 (51) | Parallel | 36 | - Median healing times - Healing rate | Unclear from study description | No | 12 weeks |
| Schuler et al. 1996 (52) | Parallel | 54 | - Complete ulcer healing - Rate of healing (‘survival’ of the nonhealing ulcer) | HCP | No | 180 days |
| Danielsen et al. 1998 (53) | Parallel | 43 | - Ulcer healing - Ulcer area reduction (mean value of ulcer duration) | HCP | Yes | 12 months  (*Equal to 48 weeks*) |
| Mosti et al. 2011(54) | Parallel | 100 | - Ulcer size - Ulcer bed appearance (presence of necrosis or fibrin, granulation tissue, epithelisation) - Skin condition: ulcer bed appearance, skin changes (scaling, blisters, redness), amount of exudate, surrounding skin condition, and ease of bandage application and removal) | Unclear from study description | Unclear from study description | 3 months |
| Smith et al. 1990 (55) | Parallel | 45 | - Complete healing of all ulcers | HCP | Unclear from study description | 3 months |
| Harding et al. 2016 (56) | Parallel | 90 | - Number of outcomes that attained complete closure - Mean duration of ulcer - Absolute reduction in ulcer size - The mean percentage reduction in ulcer size | Unclear from study description | No | 12 weeks |
| Wong et al. 2012 (57) and Wong et al. 2012 (58) and So et al. 2014 (59) | Parallel | 276 | - Time to ulcer healing - Reduction of ulcer | Unclear from study description | Yes | 24 weeks |
| Nelson et al. 2007 (60) | Factorial | 245 | - Time to completed healing - Proportion of patients healed | HCP | No | 24 weeks |
| Blecken et al. 2005 (61) | Parallel | 12 patients; 24 limbs (unit of randomisation) | - Ulcer area reduction (cm^2^ per week) - Healing rate: (initial ulcer area - final ulcer area)/(number of weeks taken to heal completely or at 12 weeks) | HCP | Unclear from study description | 12 weeks |
| NCT00821431 (62) | Parallel | 90 | - Healing: number of subjects healed | Unclear from study description | Unclear from study description | 12 weeks |
| NCT01079299 (63) | Parallel | 52 | - Time to complete wound closure (median) | Unclear from study description | No | 9 months |
| Lazareth et al. 2012 (64) | Parallel | 187 | - Complete closure - Absolute wound area reduction (cm^2^) - Relative wound area reduction (percentage) - Relative wound area reduction ≥40% *versus* baseline by week 4 - Time taken to reach complete re-epithelialisation | HCP | Yes | 12 weeks |
| Moffat et al. 2016 (65) | Parallel | 232 | - Complete ulcer closure - Time to reach complete ulcer closure - Percentage of patients with a reduction of 40% of the leg ulcer area at the last available evaluation compared to baseline - Time required to reach this percentage reduction of 40% - Absolute leg ulcer surface area reduction (area _t_0 - area _t_last) - Complete ulcer closure of the studied ulcerated limb - Complete ulcer closure of all ulcers located both in studied and contralateral limbs | Unclear from study description | Unclear from study description | 12 weeks |
| Weller et al. 2010 (66) | Parallel | 46 | - Percentage reduction of wound area | Unclear from study description | Yes | 12 weeks |
| Dolibog et al. 2013 (67) | Parallel | 70 | - Proportion of wounds healed (number of patients) - Wound dimension: average wound size - Total wound surface area (percentage) | HCP | Unclear from study description | 15 days |
| Alvarez et al. 2020 (68) | Parallel | 52 | - Median time to wound closure/complete healing - Rate of healing/speed of healing | Unclear from study description | Unclear from study description | 96 weeks |
| Arundel et al. 2023 (69) ISRCTN67321719 (70) and  Zahra 2022 (71) | Parallel | 450 | - Time to healing - Healing of the reference leg | HCP | Yes (time to healing);  No (healing of the reference leg) | 12 months |
| Cordova et al. 2022 (72) and NCT04703569 (73) | Parallel | 100 | - Size of the venous ulcer - The characteristics and clinical evolution of the venous ulcer | HCP | Yes | 26 weeks |
| Jaiswal et al. 2023 (74) | Parallel | 61 | - Decrease in ulcer size (cm) | Unclear from study description | No | 6 months |
| ISRCTN37496076 (75) | Parallel | 92 | - Ulcer wound status (healed or not healed) - Ulcer wound size | Unclear from study description | Unclear from study description | 12 weeks |
| Marston et al. 2020 (76) | Parallel | 52 | - Difference in wound area reduction from baseline: percentage reduction in wound size - Difference in wound area reduction from baseline: complete healing - Difference in wound area reduction from baseline: time to healing | Unclear from study description | No | 16 weeks |

HCP = healthcare professional; NR = not reported.

**References:**

1.	Ashby RL, Gabe R, Ali S, Saramago P, Chuang LH, Adderley U, et al. VenUS IV (Venous leg Ulcer Study IV) - compression hosiery compared with compression bandaging in the treatment of venous leg ulcers: a randomised controlled trial, mixed-treatment comparison and decision-analytic model. Health Technol Assess. 2014;18(57):1-293, v-vi.

2.	Moody M. Comparison of Rosidal K and SurePress in the treatment of venous leg ulcers. Br J Nurs. 1999;8(6):345-55.

3.	Folguera-Alvarez C, Garrido-Elustondo S, Verdu-Soriano J, Garcia-Garcia-Alcala D, Sanchez-Hernandez M, Torres-de Castro OG, et al. ECAMulticapa: Effectiveness of double-layered compression therapy for healing venous ulcers in primary care: a Study Protocol. BMC Nurs. 2016;15:58.

4.	NCT00534937. Flexitouch Compression System for Venous Stasis Ulcer 2007 [Available from: <https://clinicaltrials.gov/ct2/show/NCT00534937>.

5.	Rowland J. Intermittent pump versus compression bandages in the treatment of venous leg ulcers. Aust N Z J Surg. 2000;70(2):110-3.

6.	NCT02680834. VLU Non-Inferiority Study Comparing a Dual Action Pneumatic Compression Device to Multi-Layer Bandaging 2016 [Available from: VLU Non-Inferiority Study Comparing a Dual Action Pneumatic Compression Device to Multi-Layer Bandaging.

7.	NCT02561013. A Clinical Study to Assess a Compression Device in Patients With Venous Leg Ulcers 2015 [Available from: <https://clinicaltrials.gov/ct2/show/NCT02561013>.

8.	Dolibog P, Franek A, Taradaj J, Dolibog P, Blaszczak E, Polak A, et al. A comparative clinical study on five types of compression therapy in patients with venous leg ulcers. Int J Med Sci. 2014;11(1):34-43.

9.	Scriven JM, Taylor LE, Wood AJ, Bell PR, Naylor AR, London NJ. A prospective randomised trial of four-layer versus short stretch compression bandages for the treatment of venous leg ulcers. Ann R Coll Surg Engl. 1998;80(3):215-20.

10.	Moffatt CJ, Edwards L, Collier M, Treadwell T, Miller M, Shafer L, et al. A randomised controlled 8-week crossover clinical evaluation of the 3M Coban 2 Layer Compression System versus Profore to evaluate the product performance in patients with venous leg ulcers. Int Wound J. 2008;5(2):267-79.

11.	Szewczyk MT, Jawien A, Cierzniakowska K, Cwajda-Bialasik J, Moscicka P. Comparison of the effectiveness of compression stockings and layer compression systems in venous ulceration treatment. Arch Med Sci. 2010;6(5):793-9.

12.	Brizzio E, Amsler F, Lun B, Blattler W. Comparison of low-strength compression stockings with bandages for the treatment of recalcitrant venous ulcers. J Vasc Surg. 2010;51(2):410-6.

13.	de Abreu AM, de Oliveira BG. A study of the Unna Boot compared with the elastic bandage in venous ulcers: a randomized clinical trial. Rev Lat Am Enfermagem. 2015;23(4):571-7.

14.	Gillet JL, Guex JJ, Allaert FA, Avouac B, Leger P, Blaise S, et al. Clinical superiority of an innovative two-component compression system versus four-component compression system in treatment of active venous leg ulcers: A randomized trial. Phlebology. 2019;34(9):611-20.

15.	Milic DJ, Zivic SS, Bogdanovic DC, Jovanovic MM, Jankovic RJ, Milosevic ZD, et al. The influence of different sub-bandage pressure values on venous leg ulcers healing when treated with compression therapy. J Vasc Surg. 2010;51(3):655-61.

16.	Weller CD, Evans SM, Staples MP, Aldons P, McNeil JJ. Randomized clinical trial of three-layer tubular bandaging system for venous leg ulcers. Wound Repair Regen. 2012;20(6):822-9.

17.	Finlayson KJ, Courtney MD, Gibb MA, O'Brien JA, Parker CN, Edwards HE. The effectiveness of a four-layer compression bandage system in comparison with Class 3 compression hosiery on healing and quality of life in patients with venous leg ulcers: a randomised controlled trial. Int Wound J. 2014;11(1):21-7.

18.	Mosti G, Mancini S, Bruni S, Serantoni S, Gazzabin L, Bucalossi M, et al. Adjustable compression wrap devices are cheaper and more effective than inelastic bandages for venous leg ulcer healing. A Multicentric Italian Randomized Clinical Experience. Phlebology. 2020;35(2):124-33.

19.	Franks PJ, Moody M, Moffatt CJ, Martin R, Blewett R, Seymour E, et al. Randomized trial of cohesive short-stretch versus four-layer bandaging in the management of venous ulceration. Wound Repair Regen. 2004;12(2):157-62.

20.	O'Brien JF, Grace PA, Perry IJ, Hannigan A, Clarke Moloney M, Burke PE. Randomized clinical trial and economic analysis of four-layer compression bandaging for venous ulcers. Br J Surg. 2003;90(7):794-8.

21.	Milic DJ, Zivic SS, Bogdanovic DC, Perisic ZD, Milosevic ZD, Jankovic RJ, et al. A randomized trial of the Tubulcus multilayer bandaging system in the treatment of extensive venous ulcers. J Vasc Surg. 2007;46(4):750-5.

22.	Junger M, Wollina U, Kohnen R, Rabe E. Efficacy and tolerability of an ulcer compression stocking for therapy of chronic venous ulcer compared with a below-knee compression bandage: results from a prospective, randomized, multicentre trial. Curr Med Res Opin. 2004;20(10):1613-23.

23.	NCT00558662. Randomized Clinical Trial (RCT) to Compare the Efficacy of Coban 2 Versus SSB in the Treatment of Venous Leg Ulcers 2007 [Available from: <https://clinicaltrials.gov/ct2/show/NCT00558662>.

24.	NCT02364921. Effectiveness of Double-layer Compression Therapy in the Healing of Chronic Venous Ulcers in Primary Health Care 2015 [Available from: Effectiveness of Double-layer Compression Therapy in the Healing of Chronic Venous Ulcers in Primary Health Care.

25.	NCT02782689. Clinical Study to Assess Efficacy and Safety of a New Compression System in the Management of Venous Leg Ulcers (COMPULCE) 2016 [Available from: <https://clinicaltrials.gov/ct2/show/NCT02782689>.

26.	NCT02790593. Juxta-CuresTM Versus Bandaging for Venous Ulcers (Juxta-Cures) 2016 [Available from: <https://clinicaltrials.gov/ct2/show/NCT02790593>.

27.	Stather P, Petty C, Langthorne H, Rayner E, Zhang J, Hayden K, et al. A randomised controlled clinical trial comparing the effectiveness of bandaging compared to the JuxtaCures device in the management of people with venous ulceration: Feasibility study. Phlebology. 2021;36(7):505-14.

28.	Polignano R, Bonadeo P, Gasbarro S, Allegra C. A randomised controlled study of four-layer compression versus Unna's Boot for venous ulcers. J Wound Care. 2004;13(1):21-4.

29.	Polignano R, Guarnera G, Bonadeo P. Evaluation of SurePress Comfort: a new compression system for the management of venous leg ulcers. J Wound Care. 2004;13(9):387-91.

30.	Junger M, Partsch H, Ramelet A, Zuccarelli F. Efficacy of a Ready-Made Tubular Compression Device Versus Short-Stretch Compression Bandages in the Treatment of Venous Leg Ulc. Wounds. 2004.

31.	Mariani F, Mattaliano V, Mosti G, Gasbarro V, Bucalossi M, Blattler W, et al. The treatment of venous leg ulcers with a specifically designed compression stocking kit: Comparison with bandaging. Phlebologie -Stuttgart. 2008;37(4):191-7.

32.	Meyer FJ, Burnand KG, Lagattolla NR, Eastham D. Randomized clinical trial comparing the efficacy of two bandaging regimens in the treatment of venous leg ulcers. Br J Surg. 2002;89(1):40-4.

33.	Ukat A, Konig M, Vanscheidt W, Munter KC. Short-stretch versus multilayer compression for venous leg ulcers: a comparison of healing rates. J Wound Care. 2003;12(4):139-43.

34.	Moffatt CJ, McCullagh L, O'Connor T, Doherty DC, Hourican C, Stevens J, et al. Randomized trial of four-layer and two-layer bandage systems in the management of chronic venous ulceration. Wound Repair Regen. 2003;11(3):166-71.

35.	Moffatt CJ, Simon DA, Franks PJ, Connolly M, Fielden S, Groarke L, et al. Randomised Trial Comparing Two Four-Layer Bandage Systems in the Management of Chronic Leg Ulceration. Phlebology. 1999;14(4):139-42.

36.	Harrison MB, Vandenkerkhof EG, Hopman WM, Graham ID, Carley ME, Nelson EA, et al. The Canadian Bandaging Trial: Evidence-informed leg ulcer care and the effectiveness of two compression technologies. BMC Nurs. 2011;10:20.

37.	NCT00202267. Community RCT of the Effectiveness of Two Compression Bandaging Technologies 2005 [Available from: <https://www.clinicaltrials.gov/ct2/show/NCT00202267>.

38.	Morrell CJ, Walters SJ, Dixon S, Collins KA, Brereton LM, Peters J, et al. Cost effectiveness of community leg ulcer clinics: randomised controlled trial. BMJ. 1998;316(7143):1487-91.

39.	DePalma RG, Kowallek D, Spence RK, Caprini JA, Nehler MR, Jensen J, et al. Comparison of Costs and Healing Rates of Two Forms of Compression in Treating Venous Ulcers. Vascular and Endovascular Surgery. 1999;33(6).

40.	Gould DJ, Campbell S, Newton H, Duffelen P, Griffin M, Harding EF. Setopress vs Elastocrepe in chronic venous ulceration. Br J Nurs. 1998;7(2):66-70, 2-3.

41.	Hendricks WM, Swallow RT. Management of statis leg ulcers with Unna' S boots versus elastic support stockings. Journal of the American Academy of Dermatology. 1985;12(1):90-8.

42.	Iglesias C, Nelson EA, Cullum NA, Torgerson DJ, Ven UST. VenUS I: a randomised controlled trial of two types of bandage for treating venous leg ulcers. Health Technol Assess. 2004;8(29):iii, 1-105.

43.	Nelson EA, Iglesias CP, Cullum N, Torgerson DJ, Ven USIc. Randomized clinical trial of four-layer and short-stretch compression bandages for venous leg ulcers (VenUS I). Br J Surg. 2004;91(10):1292-9.

44.	Nikolovska S, Arsovski A, Damevska K, Gocev G, Pavlova L. Evaluation of two different intermittent pneumatic compression cycle settings in the healing of venous ulcers: a randomized trial. Med Sci Monit. 2005;11(7):CR337-43.

45.	McCulloch JM, Marler KC, Neal MB, Phifer TJ. Intermittent pneumatic compression improves venous ulcer healing. Adv Wound Care. 1994;7(4):22-4, 6.

46.	Taradaj J, Franek A, Brzezinska-Wcislo L, Blaszczak E, Polak A. Randomized trial of medical compression stockings versus two-layer short-stretch bandaging in the management of venous leg ulcers. Phlebologie. 2009;38(4):157-63.

47.	Partsch H, Damstra RJ, Tazelaar DJ, Schuller-Petrovic S, Velders AJ, de Rooij MJ, et al. Multicentre, randomised controlled trial of four-layer bandaging versus short-stretch bandaging in the treatment of venous leg ulcers. Vasa. 2001;30(2):108-13.

48.	Harley J, Harcourt D, Hutchinson B, McLean M, Long M. A comparative trial of long stretch compression bandaging versus multi-layer compression bandaging in the treatment of chronic venous ulcers. Primary Intention. 2004;12(1):6-13.

49.	Travers JP, Dalziel KL, Makin GS. Assessment of a New One-Layer Adhesive Bandaging Method in Maintaining Prolonged Limb Compression and Effects on Venous Ulcer Healing. Phlebology. 1992;7(2).

50.	Wilkinson E, Buttfieid S, Cooper S, Young E. Trial of two bandaging systems tor chronic venous leg ulcers. Journal of Wound Care. 1997;6(7):339-40.

51.	Taylor AD, Taylor RJ, Marcuson RW. Prospective Comparison of Healing Rates and Therapy Costs for Conventional and Four-Layer High-Compression Bandaging Treatments of Venous Leg Ulcers. Phlebology. 1998;13(1):20-4.

52.	Schuler JJ, Maibenco T, Megerman J, Ware M, Montalvo J. Treatment of Chronic Venous Ulcers Using Sequential Gradient Intermittent Pneumatic Compression. Phlebology. 1996;11(3):111-6.

53.	Danielsen L, Madsen SM, Henriksen L. Venous Leg Ulcer Healing: A Randomized Prospective Study of Long-Stretch versus Short-Stretch Compression Bandages. Phlebology. 1998;13(2):59-63.

54.	Mosti G, Crespi A, Mattaliano V. Comparison Between a New, Two-component Compression System With Zinc Paste Bandages for Leg Ulcer Healing: A Prospective, Multicenter, Randomized, Controlled Trial Monitoring Sub-bandage Pressures. Wounds. 2011;23(5):126-34.

55.	Smith PC, Sarin S, Hasty J, Scurr JH. Sequential gradient pneumatic compression enhances venous ulcer healing: a randomized trial. Surgery. 1990;108(5):871-5.

56.	Harding KG, Vanscheidt W, Partsch H, Caprini JA, Comerota AJ. Adaptive compression therapy for venous leg ulcers: a clinically effective, patient-centred approach. Int Wound J. 2016;13(3):317-25.

57.	Wong IK, Andriessen A, Charles HE, Thompson D, Lee DT, So WK, et al. Randomized controlled trial comparing treatment outcome of two compression bandaging systems and standard care without compression in patients with venous leg ulcers. J Eur Acad Dermatol Venereol. 2012;26(1):102-10.

58.	Wong KW, A. Andriessen A, Lee D, Thompson D, Wong L, Chao D, et al. Randomized controlled trial comparing treatment outcome of two compression bandaging systems and standard care without compression in patients with venous leg ulcers. Journal of vascular surgery. 2012;55(5):1376-85.

59.	So WK, Wong IK, Lee DT, Thompson DR, Lau YW, Chao DV, et al. Effect of compression bandaging on wound healing and psychosocial outcomes in older people with venous ulcers: a randomised controlled trial. Hong Kong Med J. 2014;20 Suppl 7:40-1.

60.	Nelson EA, Prescott RJ, Harper DR, Gibson B, Brown D, Ruckley CV. A factorial, randomized trial of pentoxifylline or placebo, four-layer or single-layer compression, and knitted viscose or hydrocolloid dressings for venous ulcers. J Vasc Surg. 2007;45(1):134-41.

61.	Blecken SR, Villavicencio JL, Kao TC. Comparison of elastic versus nonelastic compression in bilateral venous ulcers: a randomized trial. J Vasc Surg. 2005;42(6):1150-5.

62.	NCT00821431. Compression Device Versus 4-layer Compression System 2009 [Available from: <https://clinicaltrials.gov/ct2/show/NCT00821431>.

63.	NCT01079299. Effect of Intermittent Pneumatic Compression on Ulcer Healing in Subjects With Secondary Lymphedema 2010 [Available from: <https://clinicaltrials.gov/ct2/show/NCT01079299>.

64.	Lazareth I, Moffatt C, Dissemond J, Lesne Padieu AS, Truchetet F, Beissert S, et al. Efficacy of two compression systems in the management of VLUs: results of a European RCT. J Wound Care. 2012;21(11):553-4, 6, 8 passim.

65.	Moffatt C. Efficacy and safety of K-Two® versus Actico® compression system in the management of venous leg ulcers: a pilot study 2016 [Available from: <https://www.isrctn.com/ISRCTN47210331>.

66.	Weller CD, Evans S, Reid CM, Wolfe R, McNeil J. Protocol for a pilot randomised controlled clinical trial to compare the effectiveness of a graduated three layer straight tubular bandaging system when compared to a standard short stretch compression bandaging system in the management of people with venous ulceration: 3VSS2008. Trials. 2010;11:26.

67.	Dolibog P, Franek A, Taradaj J, Polak A, Dolibog P, Blaszczak E, et al. A randomized, controlled clinical pilot study comparing three types of compression therapy to treat venous leg ulcers in patients with superficial and/or segmental deep venous reflux . Ostomy Wound Manage. 2013;59(8):22-30.

68.	Alvarez OM, Markowitz L, Parker R, Wendelken ME. Faster Healing and a Lower Rate of Recurrence of Venous Ulcers Treated With Intermittent Pneumatic Compression: Results of a Randomized Controlled Trial. Eplasty. 2020;20:e6.

69.	Arundel C, Welch C, Saramago P, Adderley U, Atkinson RA, Chetter I, et al. A randomised controlled trial of compression therapies for the treatment of venous leg ulcers (VenUS 6): study protocol for a pragmatic, multicentre, parallel-group, three-arm randomised controlled trial. Trials. 2023;24(357).

70.	ISRCTN67321719. Compression therapies for the treatment of venous leg ulcers 2025 [Available from: <https://www.isrctn.com/ISRCTNISRCTN67321719>.

71.	Zahra S, Arundel C, Jones K, Davill T, Roberts G, Dumville J. VenUS 6 -- A randomised controlled trial of compression therapies for the treatment of venous leg ulcers: study design and update. Journal of Wound Management. 2022;23(1).

72.	Cordova F, Fuhrmann A, de Oliveira Machado D, Mocellin D, Uuritz da Silva B, de Fatima Lucena A, et al. Clinical and economic analysis on compression treatment of venous leg ulcers: clinical trial protocol VENOS. Eplasty. 2022;20:e6.

73.	NCT04703569. Juxta-CuresTM Versus Bandaging for Venous Ulcers (Juxta-Cures) 2016 [Available from: <https://clinicaltrials.gov/ct2/show/NCT02790593>.

74.	Jaiswal A, Pal A, Singh D, Kushwaha J, Singh K, Yadav S. Role of Four Layer Compressive Bandages in Quality of Life and Therapeutic Outcomes After Surgery for The Varicose Veins having Chronic Venous Ulcer: a Randomised Study. European Journal of Cardiovascular Medicine. 2023;13(2).

75.	ISRCTN37496076. Clinical trial of reduced leg compression for the treatment of leg ulcers, a randomised controlled trial 2023 [Available from: <https://www.isrctn.com/ISRCTN37496076>.

76.	Marston WA, Kirsner RS, Tallis A, Hanft JR, Walters J, Farber A, et al. Economic benefit of a novel dual-mode ambulatory compression device for treatment of chronic venous leg ulcers in a randomized clinical trial. J Vasc Surg Venous Lymphat Disord. 2020;8(6):1031-40 e1.
